# Splenic proteome profiling in response to Marek’s disease virus strain GX0101 infection

**DOI:** 10.1186/s12917-023-03852-5

**Published:** 2024-01-05

**Authors:** Chuan Wang, Yuanzi Liu, Yuze Yang, Man Teng, Xuerui Wan, Zixiang Wu, Zhao Zhang

**Affiliations:** 1 College of Veterinary Medicine, Gansu Agricultural University, Lanzhou, 730070 PR China; 2 Shaanxi Meili-OH Animal Health Co., Ltd, Xi’an, 712034 PR China; 3 Beijing Animal Husbandry Station, Beijing, 100107 PR China; 4 Key Laboratory of Animal Immunology of the Ministry of Agriculture, Henan Provincial Key Laboratory of Animal Immunology, Henan Academy of Agricultural Sciences, Zhengzhou, 450002 PR China

**Keywords:** Marek’s disease, GX0101, Chicken, Proteomics, Label-free technique

## Abstract

Marek’s disease virus (MDV) strain GX0101 was the first reported field strain of recombinant gallid herpesvirus type 2 (GaHV-2). However, the splenic proteome of MDV-infected chickens remains unclear. In this study, a total of 28 1-day-old SPF chickens were intraperitoneally injected with chicken embryo fibroblast (CEF) containing 2000 PFU GX0101. Additionally, a control group, consisting of four one-day-old SPF chickens, received intraperitoneal equal doses of CEF. Blood and various tissue samples were collected at different intervals (7, 14, 21, 30, 45, 60, and 90 days post-infection; dpi) for histopathological, real-time PCR, and label-free quantitative analyses. The results showed that the serum expressions of MDV-related genes, *meq* and *gB,* peaked at 45 dpi. The heart, liver, and spleen were dissected at 30 and 45 dpi, and their hematoxylin-eosin staining indicated that virus infection compromised the normal organizational structure at 45 dpi. Particularly, the spleen structure was severely damaged, and the lymphocytes in the white medulla were significantly reduced. Furthermore, liquid chromatography-mass spectrometry (LC-MS) and label-free techniques were used to analyze the difference in splenic proteome profiles of the experimental and control groups at 30 and 45 dpi. Proteomic analysis identified 1660 and 1244 differentially expressed proteins (DEPs) at 30 and 40 dpi, respectively, compared with the uninfected spleen tissues. According to GO analysis, these DEPs were involved in processes such as organelle organization, cellular component biogenesis, cellular component assembly, anion binding, small molecule binding, metal ion binding, cation binding, cytosol, nuclear part, etc. Additionally, KEGG analysis indicated that the following pathways were linked to MDV-induced inflammation, apoptosis, and tumor: Wnt, Hippo, AMPK, cAMP, Notch, TGF-β, PI3K-Akt, Rap1, Ras, Calcium, NF-κB, PPAR, cGMP-PKG, Apoptosis, VEGF, mTOR, FoxO, TNF, JAK-STAT, MAPK, Prion disease, T cell receptor, and B cell receptor. We finally screened 674 DEPs that were linked to MDV infection in spleen tissue. This study improves our understanding of the MDV response mechanism in the spleen.

**Supplementary Information:**

The online version contains supplementary material available at 10.1186/s12917-023-03852-5.

## Introduction

Marek’s Disease (MD), a serious threat to chicken health, causes huge economic losses to the poultry industry worldwide [1, 2]. It is an immunosuppressive malignant tumor disease, characterized by increased T lymphocytes, and is caused by the infection of MDV in chickens [3]. The MD leads to diverse physiological dysfunctions in chickens, typically including the following five: 1) immunosuppression and lymphoma formation in the lymphatic system; 2) atherosclerotic injury of the cardiovascular system; 3) various peripheral and central nervous system lesions; 4) formation of sporadic or diffuse tumors of different sizes in the viscera with destruction of normal tissue structure; and 5) epithelium injury and the formation of skin feather sac knots. MDV infection induces tumors and immune organ atrophy in the host, resulting in immune suppression [4, 5].

The natural infection process of MDV has four major stages: early cytolytic infection, latent infection, late cytolytic infection, and transformational infection [6]. *Meq* and *gB* (glycoprotein B) are the two MDV-related genes; *meq* directly participates in the development of MD tumors, while *gB* is the most conserved structural gene of MDV. *gB* encodes a group of glycoprotein complexes containing eight glycosylation sites [7]. gB is located on the surface of the infected cell membrane and cytoplasm, promoting humoral and cellular immunity in the host, and therefore it is the main neutralizing antigen of MDV. gB proteins mainly participate in virus adsorption and invasion into host cells [8].

Past research has studied the host protein expression at different stages of MDV infection in chicken immune organs by two-dimensional electrophoresis (2-DE) and mass spectrometry (MS). For example, chickens were infected with the MDV RB1B strain in the bursa of Fabricius and spleen to examine transcriptome change post-infection [9, 10]. Another study examined the splenic proteome of chickens infected with the MDV JM-16 strain [11]. MDV Chinese strain GX0101 was isolated in 2001 from a vaccinated flock of layer chickens with severe tumors. It was the first reported field strain of recombinant gallid herpesvirus type 2 (GaHV-2) [12]. Reticuloendotheliosis virus (REV) long terminal repeat (LTR) insert promoted horizontal transmission of MDV strain GX0101 compared to other viral strains [13, 14]. GX0101 is a very virulent MDV (vvMDV) with higher horizontal transmission ability than the other vvMDV strain, Md5. The complete genome sequence of GX0101 is 178,101 nucleotides [15]. The *meq* deletion mutant of GX0101 showed significantly reduced immunosuppression in chickens [16]. Although the changes in the splenic transcriptome of GX0101-infected chickens during early infection and pathogenic phases have been determined by RNA-seq [17], the corresponding changes in the splenic proteome remain unclear. In this study, we used label-free quantitative proteomics technology to analyze the splenic proteome of GX0101-infected chickens. Based on the involved pathways of inflammation, apoptosis, and tumor in MDV infection, we screened for related signaling pathways and differential proteins (DEPs) in the chicken spleen after 30 and 45 days post-infection (dpi).

## Materials and methods

### Animal experiments and sample preparation

MDV GX0101 was donated by the College of Veterinary Medicine, Shandong Agricultural University, China. The animal experiments were conducted following the protocols of the Ethical and Animal Welfare Committee of the Key Laboratory of Animal Immunology, Ministry of Agriculture, China [18]. SPF chicken embryos were purchased from Zhengzhou Ruixiang Hatchery, China. A total of 32 one-day-old SPF chickens were divided into the control (*n* = 4) and experimental (*n* = 28) groups. Additionally, the experimental group had 7 sub-groups (*n* = 4). Each chick in the experimental group was intraperitoneally injected with chicken embryo fibroblasts (CEFs) containing 2000 PFU GX0101, while control chickens were injected with equal doses of CEF cultures. The chickens in the experimental and control groups were fed in two SPF isolators with the same environmental conditions, and their deaths and mental states were recorded. According to the 2020 edition of the American Veterinary Medical Association Guidelines for the Euthanasia of Animals, chickens were anesthetized intravenously with sodium pentobarbital (30 mg/kg) and then exsanguinated to death at 7, 14, 21, 30, 45, 60, and 90 dpi, respectively. All blood and tissue samples from the heart, liver, spleen, lung, kidney, and glandular stomach tissues were collected in sterile tubes, snap-frozen in liquid nitrogen, and then stored at − 80 °C for further use. These samples were examined for histopathology, real-time PCR, and label-free quantitative proteomics. The histological samples were fixed in 4% neutral paraformaldehyde for more than 15 days.

### DNA extraction and viral load estimation by qPCR

The animal blood samples were collected at 7, 14, 21, 30, 45, 60, and 90 dpi in the experimental groups. The viral genome was extracted from blood using the Whole Blood Genomic DNA Extraction Kit (TransGen, China). The *meq* and *gB* recombinant plasmids were constructed. The absolute quantitative standard curves of *meq* and *gB* were established by qPCR by comparing levels with standard plasmids as described previously [19]. The serum expression levels of *meq* and *gB* were detected by qPCR and, the data were analyzed by the absolute quantitative method. qPCR was performed on a LightCycler 96 (Roche, Switzerland) instrument, using water as a negative control. Each reaction volume was 20 μL, including 10 μL SYBR Premix Ex TaqTM (Vazyme, China), 0.5 μL forward primer (10 mM), 0.5 μL reverse primer (10 mM), 1.0 μL DNA template and 8 μL ddH_2_O. The primer sequences used are listed in Table 1. The temperature program was as follows: 95 °C for 120 s, 95 °C for 15 s, 60 °C for 60 s (35 cycles), and 72 °C for 20 s. Triplicate datasets were analyzed using LightCycler® 96 software (version 1.1.0.1320).

**Table 1:** The sequences of primers used in this study

Name	Primer sequence(5′-3′)	Size	NCBI No.
*meq*	Sense: ACGCTCAGCTTTGTCCTGTT	181	JX844666.1
	Antisense: GGAAACCACCAGACCGTAGA		
*gB*	Sense: CCGCTCTGTGTTTCCGTATT	191	JX844666.1
	Antisense: TGACTGGAAGGCTTGCTTTT		
*GAPDH*	Sense: TGGGTGTCAACCATGAGAAA	171	NM_204305.2
	Antisense: CATCCACCGTCTTCTGTGTG		
*PRNP*	Sense: ACCGATGGTGGAGTGAGAAC	221	GU991271.1
	Antisense: GGATCACCTTCGTCACCACT		
*IFNLR1*	Sense: AGCCGGATCTGAAGACAAGA	156	NM_001389541.2
	Antisense: CACACTGGCTGGGAGAATTT		
*FN1*	Sense: TTTGGGTATGCAGTGGTTGA	159	NM_001198712.2
	Antisense: GTCCTCCCGTTGTAGGTGAA		
*CTSD*	Sense: CCAAGGAAGTGAAGGAGCTG	190	NM_205177.2
	Antisense: CTCAGGCAGATGGTCTCTCC		
*CD79B*	Sense: ACGGGAACAGCACCAGTAAC	170	NM_001006328.3
	Antisense: CACGTGGAACTCCTTTCCAT		
*IL2RA*	Sense: CGAAGCAAGCAAACAATTCA	169	NM_204596.2
	Antisense: TCCACATTCTTGCACGTGAT		
*MAVS*	Sense: GGGACATCCAGCACAGTTTT	174	AB772011.1
	Antisense: AGCACTCAAATCCCTTGGTG		
*STAT1*	Sense: CAGATGGAAGTGGGAGGTGT	208	NM_001012914.2
	Antisense: CCTCTTGTGATGCACCATTG		
*STAT3*	Sense: TTGGAACAGATGCTCACAGC	184	NM_001030931.2
	Antisense: TCAAGCCGGTCTAAGCAGAT		

### Histopathology

The heart, liver, spleen, lung, kidney, and glandular stomach tissues were fixed in a 4% formaldehyde solution to make paraffin-embedded sections of 5 μm. The tissue sections were stained with hematoxylin and eosin and examined under a light microscope (Leica DM750) as described previously [20].

### Protein extraction, reductive alkylation, trypsin digestion, and LC-MS/MS analysis

Proteins were extracted from respective spleen tissues at 30 dpi and 45 dpi for crude analysis. The four duplicate samples in each group were mixed up. All samples were transferred to a centrifuge tube and centrifuged at 10,000×g for 30 min at 4 °C. Lysis buffer was then added to extract total proteins. The proteins were precipitated with trichloroacetic acid and centrifuged at 40,000×g for 30 min at 4 °C. The protein concentration was determined using the Qubit fluorescent protein quantification kit (Invitrogen, China). The processing of protein samples and LC-MS/MS analysis were performed as described previously [21].

### Differential expression analysis

Tandem mass spectra were searched against Mascot 2.1 (local host) chickens protein databases. The search results were filtered using a cutoff of 1% peptide false identification rate (Peptide FDR). The peptides with a Z score < 4 or a delta-mass > 5 ppm were rejected. Additionally, the minimum number of peptides needed to identify a protein was set to 1. We used the Quantitative Software Profile Analysis 2.0 program with default parameters for data analysis. DEPs were screened with a threshold of 1.5-fold change (FC = fold change). DEPs with FC ≥1.5 were considered upregulated, those with FC ≤ 0.667 were downregulated, and those with 0.667< FC <1.5 were deemed to have no significant change in their levels.

### Gene ontology (GO) and Kyoto encyclopedia of genes and genomes (KEGG) enrichment analyses of DEPs

GO analysis of DEPs was performed through the Evolutionary Relationships (PANTHER) database version 6.1 (www.pantherdb.org). The GO enrichment analysis first mapped all DEPs to each term of the GO database (http://www.geneontology.org/) and then calculated the number of proteins per term. After that, it applied a hypergeometric test to identify GO entries that were significantly enriched in DEPs compared with the background proteins, and their *P*-values were calculated. GO terms with a corrected *P* value ≤0.05 were considered significantly enriched by DEPs.

Signaling pathway analysis was performed with the KEGG database (http://www.genome.jp/kegg/pathway.html), similar to the GO enrichment analysis. Pathways with a *P*-value ≤0.05 were considered significantly enriched by DEPs.

### Protein-protein interaction networks (PPI) and clustering analysis of DEPs

The PPI networks (confidence score ≥ 0.40) of DEPs were obtained using the STRING database (https://string-db.org/) and visualized by Cytoscape software (v3.7.2) to perform the clustering analysis as described previously [22].

### Real-time PCR

Total RNA was extracted from the spleen tissues using the Ultrapure RNA Kit, including DNase I (ComWin, Beijing, China). The cDNA was synthesized using the TransScript First-Strand cDNA Synthesis SuperMix kit (TransGen, Beijing, China) following the manufacturer’s protocol. Primers were designed by Prime 5.0 software based on the gene sequences from the NCBI (Table 1). Reaction volume, procedures, instruments, and software were similar to those above mentioned for qPCR. The relative expression values of genes were calculated using the 2^-ΔΔCt^ method and normalized to GAPDH*,* an internal reference gene [23].

### Statistical analysis

Statistical analysis was performed using IBM SPSS, version 25.0 (Chicago, IL). A one-way analysis of variance (ANOVA) was used to detect statistical differences between the control and the experimental groups.

## Results

### Serum viral load at different periods of infection

**Fig. 1:**
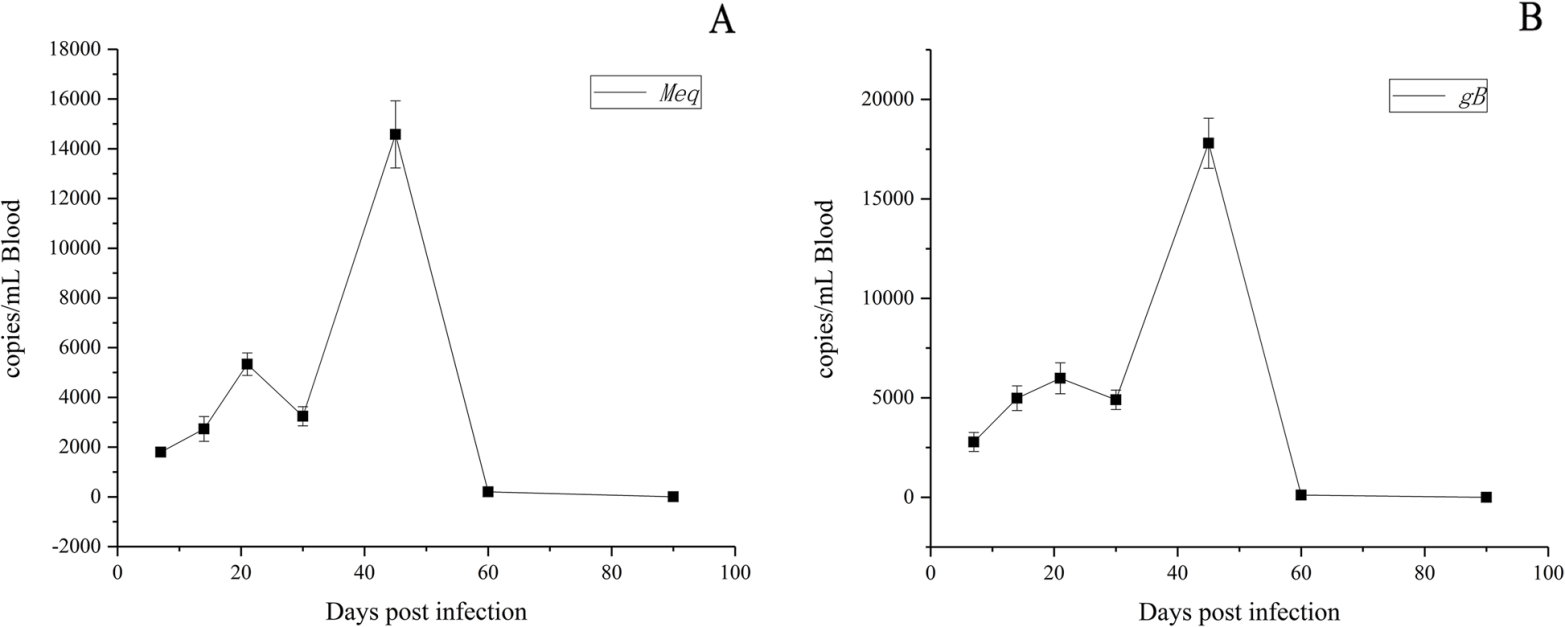
The qPCR detection of *meq* and *gB* gene expressions in chicken blood after MDV infection. The change in serum expression of (**A**) *meq* and (**B**) *gB* after MDV infections

As shown in Fig. 1A, B, both *gB* and *meq* exhibited similar expression trends, which peaked at 21 dpi after the GX0101 infection. Subsequently, their levels slumped to the bottom at 30 dpi and then rapidly increased to the maximum at 45 dpi. From thereon, the expression level gradually decreased to 90 dpi and remained at a low level.

### Pathological manifestations of organs in infected chickens

**Fig. 2:**
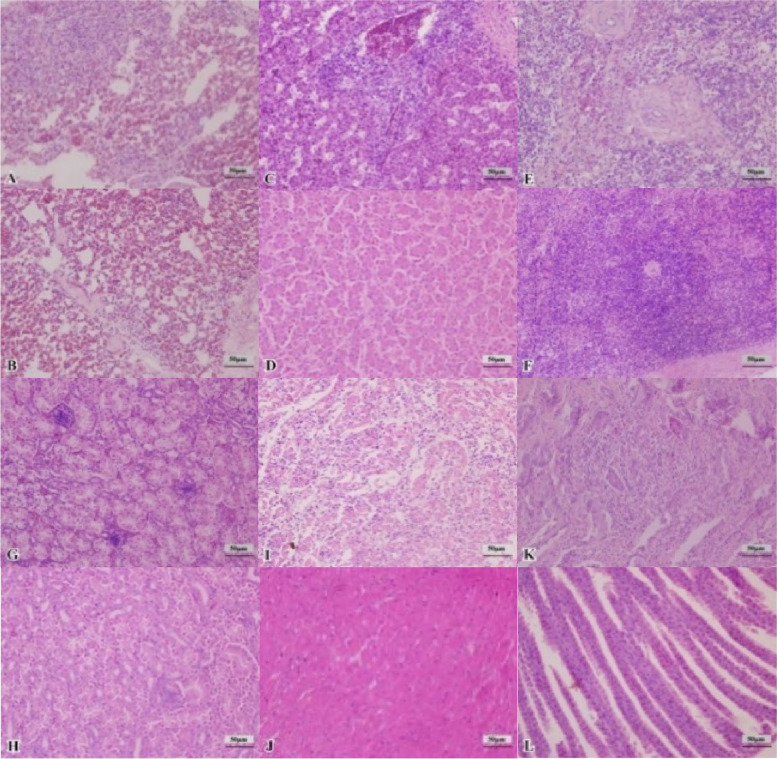
Histopathologic observation of chickens (400x) at 45 dpi. **A**, **C**, **E**, **G**, **I**, and **K**: Pathological observation of lung, liver, spleen, kidney, heart, and glandular stomach in MDV-infected tumor-diseased chickens. **B**, **D**, **F**, **H**, **J**, and **L**: Pathological observation of lung, liver, spleen, kidney, heart, and glandular stomach in the healthy control group

The microscopical images showing the histopathology of tissue sections are shown in Fig. 2. Compared with the control group, the normal organizational structure of the internal organs in experimental chickens was compromised by tumor tissue at 45 dpi. Neoplastic cells exhibited pleomorphism with darker cytoplasmic staining, less cytoplasm, heteromorphic nuclei, a low degree of differentiation, and a visible abnormal mitotic phase. The liver demonstrated local congestion, diffuse tumor cell proliferation, hepatocyte cord swelling, hepatic sinusoidal atrophy, and local lymphocyte proliferation. The lungs had local congestion and hemorrhage, widening of the pulmonary interstitium, and significant lymphocyte proliferation, accompanied by tumor cell proliferation. The spleen structure was severely damaged, the white pulp lymphocytes were significantly reduced, and there were some necrotic cells. The kidney showed interstitial congestion and renal tubular epithelial cells were degenerated and necrotized. Also, the renal interstitium had tumor cell proliferation. The heart showed clear lesions with tumor cell proliferation along with myocardial fibrosis and inflammatory cell infiltration. The myocardial membrane was ruptured with interstitial edema. The glandular stomach exhibited severe lymphocyte proliferation in the lamina propria, accompanied by tumor cell hyperplasia and the destruction of the original structure. Meanwhile, no obvious histopathological changes were observed in the liver, spleen, and kidney at 30 dpi in the experimental group as in the control group.

### Analysis of total DEPs

**Fig. 3:**
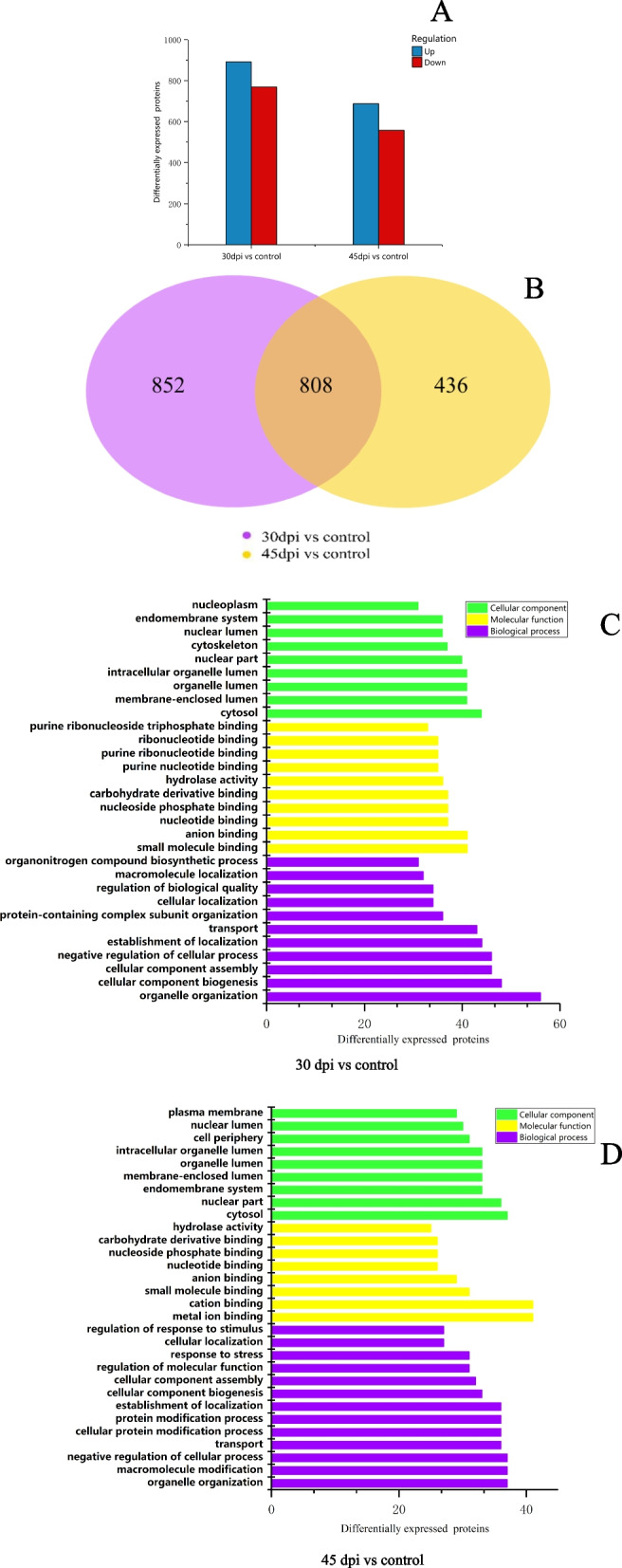
Proteomic data analysis. **A** Protein expression differences of MDV-infected chickens at 30 and 45 dpi. Fold Change > 1.5 and a Fold Change < 0.667 was the threshold. The blue plots represent upregulated DEPs (Fold Change > 1.5) and the red plots represent downregulated DEPs (Fold Change < 0.667). **B **Venn diagram of total DEPs. Purple represents the 30 dpi group. Yellow represents the 45 dpi group. GO functional enrichment pathway of (**C**) 30 dpi and (**D**) 45 dpi

In this study, we selected DEPs based on FC > 1.5 with a *P*-value ≤0.05. Compared with the control group, in total, 2904 DEPs were identified in both 30 and 45 dpi samples. Splenic proteomic analysis revealed 1660 (891 upregulated and 769 downregulated) and 1244 (687 upregulated and 557 downregulated) DEPs at 30 and 45 dpi, respectively, compared with the uninfected spleen tissues (Fig. 3A). Among the DEPs, 852 and 436 were unique to 30 and 45 dpi, respectively. Meanwhile, there were 808 shared DEPs between 30 and 45 dpi samples (Fig. 3B).

### GO analysis of DEPs

To fully analyze the potential cellular function associated with DEPs, we performed GO functional enrichment analysis (Fig. 3C, D). The DEPs were mainly involved in intracellular molecular and ion binding, cellular component organization, cellular processes, and biological regulation.

### KEGG enrichment analysis

**Fig. 4:**
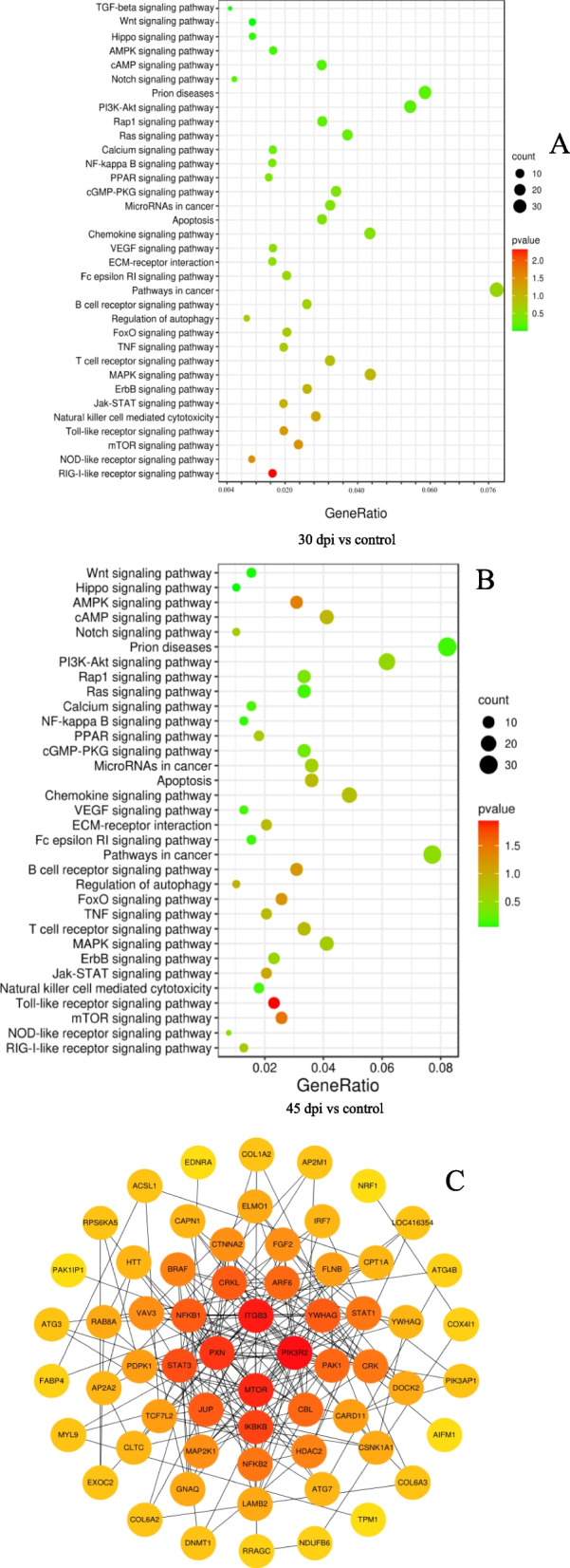
Enrichment analysis of selected DEPs in the splenic proteome of GX01010-infected chickens. KEGG enrichment analysis of selected DEPs in (**A**) 30 dpi and (**B**) 45 dpi groups. **C** The protein−protein interaction (PPI) networks of DEPs at the 30 and 45 dpi in the KEGG pathway

To explore the KEGG pathways linked to inflammation, apoptosis, and tumors, we screened 34 relevant signaling pathways related to DEPs identified at 30 dpi and 33 relevant signaling pathways related to DEPs identified at 45 dpi (Fig. 4A, B). These pathways included the Toll-like receptor, mTOR, AMPK, FoxO, B cell receptor, Jak-STAT, cAMP, Apoptosis, T cell receptor, TNF, Chemokine, PPAR, MAPK, Notch, PI3K-Akt, ErbB, Rap1, cGMP-PKG, VEGF, Calcium, Wnt, NF-κB, Ras, Hippo, Prion diseases, and Fc epsilon RI signaling pathway. The TGF-beta pathway was associated only with DEPs identified in 30 dpi samples.

### PPI network and clustering analyses of DEPs

We observed that different DEPs were in the same signal pathway (Table 2). There were 83 shared DEPs in the signal pathways of the 30 dpi and 45 dpi experimental groups. A PPI network of 83 DEPs, with 62 nodes and 191 edges, was constructed using the STRING database and visualized by Cytoscape (v3.7.2) (Fig. 4C). In the PPI network, the top hub proteins were *ITGB3*, *PIK3R2*, *MTOR*, *PXN*, *I*κ*B*, *JUP*, *STAT3*, *NFKB1*, *CRKL,* and *ARF6*.

**Table 2:** KEGG pathway enrichment analysis of DEPs identified at 30 dpi and 45 dpi of MDV

KEGG pathway	Common proteins	Specific proteins	
		30 dpi specific proteins	45 dpi specific proteins
RIG-I-like receptor signaling pathway	IRF7/NFKB1/NLRX1/ IκBKB/ RELA /	TRIM25/MAVS/ ATG5	
NOD-like receptor signaling pathway	NFKB1/ IκBKB/ RELA /	FNIP1/MAPK1	
mTOR signaling pathway	IκBKB/MTOR/PIK3R2/ PDPK1/CAB39/RRAGC/ BRAF/	PRKCA/RPS6KA1/ MAPK1	PRKAA1/RICTOR/
Natural killer cell mediated cytotoxicity	PIK3R2/BRAF/ MAP2K1/PPP3R1/ VAV3/PAK1/	PTPN11/LOC419429/ BF1/ MAPK1/ PRKCA/	
Jak-STAT signaling pathway	MTOR/PIK3R2/STAT1/ STAT3/	STAT5B/STAM2/ PTPN11/ LOC419429/	CNTFR/IFNLR1/ IL2RA/
MAPK signaling pathway	NFKB1/ IκBKB/ RELA / BRAF/MAP2K1/ PPP3R1/PAK1/CRK/ CRKL/RPS6KA5/ NFKB2/FLNB/FGF2/	PLA2G4A/LAMTOR3/STMN1/ATF2/MAPK1/PRKCA/ RPS6KA1	MAP2K6/ RRAS2/ RAP1B/
T cell receptor signaling pathway	NFKB1/ IκBKB/ RELA / PIK3R2/PDPK1/ MAP2K1/PPP3R1/ VAV3/PAK1/CBL/ CARD11/	PRKCQ/MAPK1	NFKBIE/
FoxO signaling pathway	IκBKB/PIK3R2/PDPK1/ BRAF/MAP2K1/STAT3/	CAT/SGK3/ MAPK1	PRKAA1/PCK2/ SOD2/
B cell receptor signaling pathway	NFKB1/ IκBKB/ RELA/PIK3R2/ MAP2K1/PPP3R1/VAV3/ CARD11/PIK3AP1/	BTK/MAPK1	NFKBIE/CD79B/
Pathways in cancer	NFKB1/ IκBKB/ RELA / MTOR/PIK3R2/BRAF/ STAT1/ MAP2K1/ STAT3/ CBL/ CRK/ CRKL/NFKB2/FGF2/ CTNNA2/HDAC2/ GNAQ/LAMB2/ LOC416354/JUP/ EDNRA/CCDC6/ TCF7L2/	RASSF5/ FNIP1/ MAPK1/PRKCA/ STAT5B/ COL4A2/ TPM3/ ITGA3/GNG5/ARNT/SLC2A1	GNAI2/ITGA2/ FN1/TCEB1/ APPL1/
VEGF signaling pathway	PIK3R2/MAP2K1/ PPP3R1/PXN/	MAPK1/PRKCA/ PLA2G4A	
ECM-receptor interaction	LAMB2/ITGB3/COL6A2/ COL6A3/COL1A2/	COL4A2/ ITGA3/ CD44	ITGA2/FN1/ THBS1/
Chemokine signaling pathway	NFKB1/ IκBKB/ RELA/ PIK3R2/BRAF/STAT1/ MAP2K1/VAV3/PAK1/ STAT3/CRK/CRKL/ PXN/DOCK2/ELMO1/	MAPK1/STAT5B/ GNG5/WASL	GNAI2/RAP1B/ XCL1/
Apoptosis	NFKB1/ IκBKB/RELA / PIK3R2/PDPK1/ MAP2K1/TUBA1C/ AIFM1/BAK1/CAPN1/	MAPK1/LMNB1/ LMNA/	TUBA3E/CTSD/ DFFA/
MicroRNAs in cancer	NFKB1/ IκBKB/MTOR/ MAP2K1/STAT3/CRK/ CRKL/RPS6KA5/ ITGB3/ BAK1/DNMT1/ TPM1/	PRKCA/STMN1/CD44	THBS1/EZR/
cGMP-PKG signaling pathway	PIK3R2/MAP2K1/ PPP3R1/GNAQ/ EDNRA/SLC25A6/ ATP1B1/MYL9/ GUCY1A2/PRKG1/	MAPK1/ATF2/SLC25A4/PPP1CB/ATP2B1	ATP1A3/GNAI2/
PPAR signaling pathway pathway	PDPK1/CPT1A/FABP4/ ACSL1/	ACAA1/ILK/CPT2	PCK2/FABP7/ FABP5/
NF-kappa B signaling pathway	NFKB1/ IκBKB/RELA/ NFKB2/CARD11/	TRIM25/ PRKCQ/ BTK	
Calcium signaling pathway	PPP3R1/GNAQ/ EDNRA/SLC25A6/ STIM1/	PRKCA/ SLC25A4/ ATP2B1	P2RX1/
Ras signaling pathway	NFKB1/ IκBKB/RELA/ PIK3R2/MAP2K1/ PAK1/FGF2/EXOC2/ ARF6/	MAPK1/ PRKCA/ PTPN11/ LOC419429/ PLA2G4A/ RASSF5/ GNG5	RRAS2/RAP1B/ RAB5B/
Rap1 signaling pathway	PIK3R2/BRAF/ MAP2K1/CRK/CRKL/ FGF2/GNAQ/ITGB3/	MAPK1/ PRKCA/ RASSF5/TLN2/	MAP2K6/ GNAI2/ RAP1B/ THBS1/
PI3K-Akt signaling pathway	NFKB1/ IκBKB/RELA/ MTOR/PIK3R2/PDPK1/ MAP2K1/FGF2/ PIK3AP1/LAMB2/ ITGB3/COL6A2/ COL6A3/COL1A2/ YWHAQ/YWHAG/	FNIP1/MAPK1 PRKCA/ATF2/SGK3/ COL4A2/ ITGA3/ GNG5	PRKAA1/ PPP2R5C/ PCK2/ IL2RA/ ITGA2/ FN1/ THBS1/
Notch signaling pathway	HDAC2/LOC416354/ SNW1/		PSEN1/
cAMP signaling pathway	NFKB1/RELA/PIK3R2/ BRAF/MAP2K1/VAV3/ PAK1/EDNRA/ATP1B1/ MYL9/	MAPK1/ PPP1CB/ ATP2B1	RRAS2/ATP1A3/ GNAI2/RAP1B/ CNGA3/
AMPK signaling pathway	MTOR/PIK3R2/PDPK1/ CAB39/CPT1A/RAB8A/ PFKM/		PRKAA1/PPP2R5C/RAB11B/PCK2/
Hippo signaling pathway	CTNNA2/TCF7L2/ YWHAQ/YWHAG/	PPP1CB	
Wnt signaling pathway	PPP3R1/LOC416354/ TCF7L2/CSNK1A1/	PRKCA	PSEN1/TBL1X/
Prion diseases	MAP2K1/PPP3R1/ HDAC2/GNAQ/CAPN1/ SLC25A6/C7/C6/ CLTC/AP2M1/ATP8/ COX4I1/NDUFB6/ DCTN2/AP2A2/HTT/ DCTN4/NRF1/POLR2F/ UBE2G1/UCHL1	MAPK1/ SLC25A4/ COX7A2/ NDUFV1/ NDUFB3/ SEP-05	SOD2/GNAI2/ PSEN1/NDUFA9/ NDUFB10/LRP1/ UBE2G2/CLTA/ TGM2/ POLR2L
Fc epsilon RI signaling pathway	PIK3R2/PDPK1/ MAP2K1/VAV3/	MAPK1/PRKCA/BTK/PLA2G4A	MAP2K6/
Regulation of autophagy	ATG7/ATG3/ATG4B/ ATG4B/	ATG5	PRKAA1/
TNF signaling pathway	NFKB1/ IκBKB/RELA/ PIK3R2/MAP2K1/ RPS6KA5/	MAPK1/ATF2/	MAP2K6/
Toll-like receptor signaling pathway	IRF7/NFKB1/ IκBKB/ RELA/PIK3R2/STAT1/ MAP2K1/	MAPK1	MAP2K6/
ErbB signaling pathway	MTOR/PIK3R2/ BRAF/ MAP2K1/PAK1/ CBL/CRK/CRKL/	MAPK1/PRKCA/ STAT5B	
TGF-beta signaling pathway		MAPK1/ E2F4	

### qPCR validation of proteomic data

**Fig. 5:**
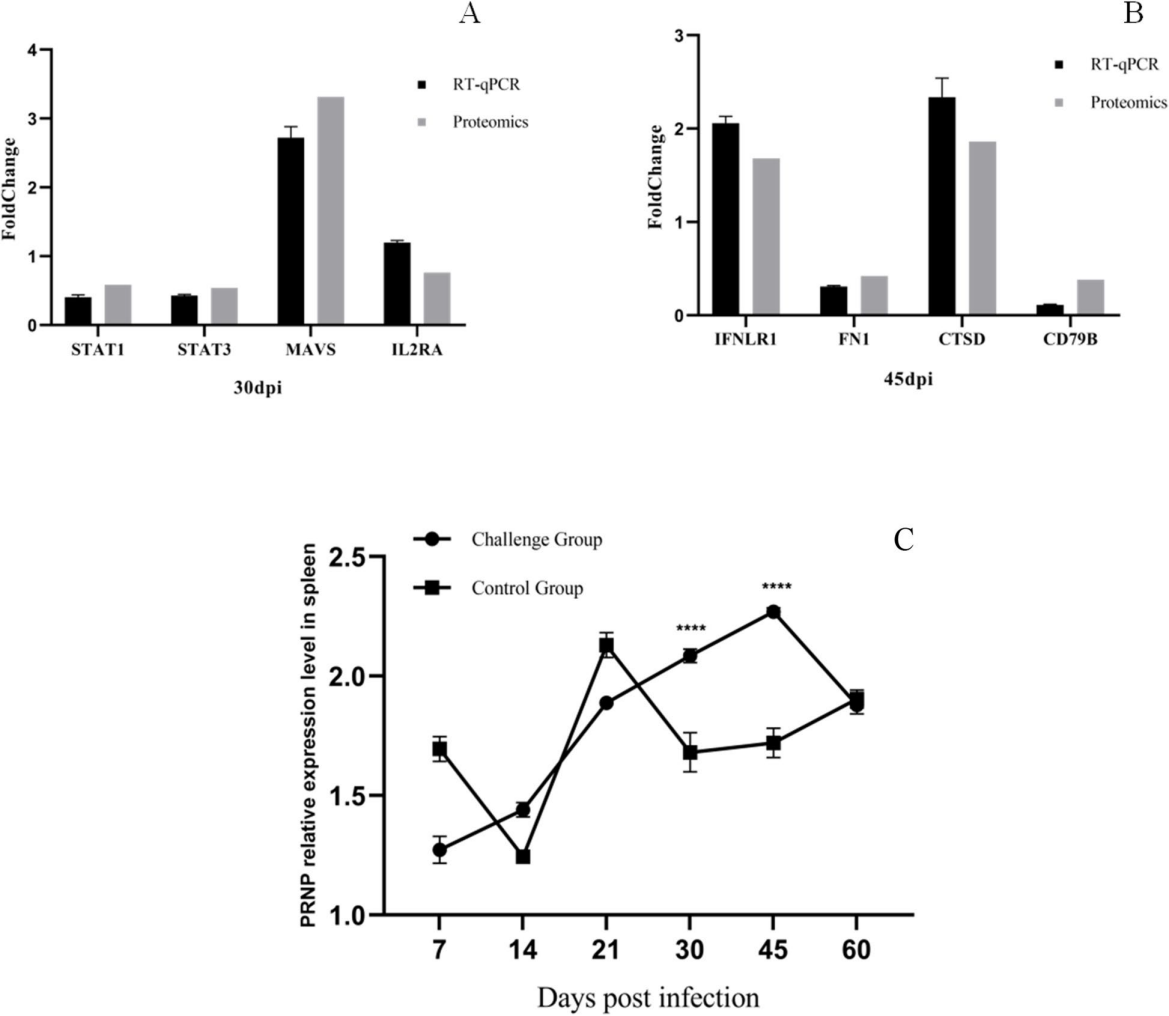
The RT-qPCR validation of DEPs. RT-qPCR validation of DEPs at (**A**) 30 dpi and (**B**) 45 dpi. (**C**) RT-qPCR detection of *PRNP* expression at 7, 14, 21, 30, 45, and 60 dpi. *GAPDH* was the internal reference gene. ***P<0.01

We used qPCR to validate the proteomic data by estimating the mRNA expressions of selected DEPs. As shown in Fig. 5A, B, the mRNA expressions of *FN1*, *CD79B*, *STAT1,* and *STAT3* were considerably downregulated, whereas those of *IFNLR1*, *CTSD,* and *MAVS* were significantly upregulated. These expression results were consistent with proteomic data. Notably, the qPCR result of *IL2RA,* i.e.*,* upregulation of *IL2RA*, was different from the proteome results. Also, the expression of *PRNP* varied with MDV infection and infection time (Fig. 5C).

## Discussion

In recent years, the emergence of targeted gene and protein expression profiles has facilitated a better understanding of host responses to MDV. Moreover, modern techniques and tools for analyzing host cell proteomes and transcriptomes have accelerated the exploration of complex interactions between the host and the pathogen. We observed that in chickens infected with GX0101, the serum load of MDV reached its peak at 45 dpi. Histopathology revealed the destruction of the normal organizational structure of the internal organs in the experimental chickens at 45 dpi, characterized by the presence of tumor tissue. Notably, the spleen structure suffered severe damage, with a significant reduction in lymphocytes in the white pulp.

We then conducted proteome analysis of spleen tissue at 30 and 45 dpi, which laid the foundation for subsequent studies on MDV strain GX0101. In this study, we identified 1660 and 1244 DEPs at 30 dpi and 45 dpi, respectively, using the label-free quantitative proteomics technology. Notably, a previous study on splenic protein expression in MDV strain RB1B-infected chickens, employing 2-DE and MS, identified only 58 DEPs [9]. Additionally, a study combining 2-DE and MS analysis of the splenic proteomics of genetically resistant and susceptible chickens infected with MDV strain JM-16 identified only 40 DEPs [11]. While several MDV strains have been analyzed for splenic proteomics, these prior studies primarily utilized 2-DE and MS. In contrast, the label-free quantitative proteomics technology employed in our study offers higher resolution and accuracy.

To investigate the mechanism of splenic tissue damage caused by MDV in chickens, we analyzed KEGG pathways related to inflammation, apoptosis, and tumor based on the proteomic results. The results revealed that the signaling pathways were consistent in both the 30 dpi and 45 dpi experimental groups, but there were differences in DEPs. The pathways linked to inflammation and apoptosis in both groups included Toll-like receptor, FoxO, B cell receptor, AMPK, regulation of autophagy, apoptosis, T cell receptor, TNF, PPAR, Notch, RIG-I-like receptor, PI3K-Akt, NOD-like receptor, Calcium, cGMP-PKG, natural killer cell-mediated cytotoxicity, NF-κB, and Fc epsilon RI pathway.

In primary lymphoid organs such as the bursa, thymus, and spleen, MDV initially replicated in B cells and then infected T cells through direct intercellular transfer, ultimately causing T-cell lymphoma [24]. This finding is consistent with our results, indicating that both the T-cell and B-cell receptor pathways play a role in altering protein expression in tumor formation. Furthermore, the pathways linked to GX0101-induced tumors in both the 30 dpi and 45 dpi experimental groups included mTOR, MAPK, cAMP, ECM-receptor interaction, Chemokine, JAK-STAT, MicroRNAs in cancer, ErbB, pathways in cancer, Rap1, VEGF, Ras, Wnt, Prion diseases, and Hippo pathway. The MAPK pathway, a tumorigenesis-related pathway that is critical for inflammation and cancer development, was also identified [25, 26]. Notably, the MAPK pathway has also been implicated in previous MD studies [27, 28].

The transcriptome analysis of the chicken infected with the RBlB strain of MDV uncovered alterations in tumor-related pathways, such as the WNT signaling pathway, JAK-Stat signaling pathway, Notch signaling pathway, MAPK signaling pathway, and TGF-Beta signaling pathway [29]. The TGF-beta signaling pathway was linked to MDV tumorigenesis [30]. Nonetheless, in our study, the TGF-beta pathway displayed DEPs at 30 dpi, but no DEP was found at 45 dpi, suggesting that the TGF-beta pathway might only be implicated in the early and middle stages of viral infection. We conducted PPI network analysis for DEPs in the selected pathway at 30 and 45 dpi. We screened several hub proteins, including ITGB3, PIK3R2, MTOR, PXN, IκB, JUP, STAT3, NFKB1, CRKL, and ARF6. The ITGB3 protein, known as the integrin beta chain beta 3, was identified as an epithelial-to-mesenchymal transition biomarker in colorectal cancer, prostate cancer, and breast cancer [31, 32]. ITGB3 influences tumor immunity through both the innate and adaptive immune systems. TGB3 could function as a regulator to enhance TGF-β/H_2_O_2_/HOCl signaling, thereby transforming non-metastatic tumors into metastatic tumors [33]. Activated macrophages exerted anti-tumor effects by producing inflammatory cytokines like IL-1β, while the interaction between ITGB3 and MFGE8 inhibited macrophage IL-1β production by inducing necrotic cells and an ATP-dependent manner [34]. The PIK3R2 protein serves as a regulatory component of PI3K, and targeting PIK3R2 could inhibit the proliferation, migration, and invasion of NSCLC A549 cells by modulating the PTEN/PI3K/AKT pathway [35]. MTOR exists in two complexes, MTORC1 and MTORC2. MTORC1 controls protein synthesis, cell growth, and proliferation, while MTORC2 regulates the actin cytoskeleton, promoting cell survival and cell cycle progression [36, 37]. MTOR plays a crucial role in tumor progression, and mTOR inhibitors have been employed in clinical cancer treatment [38]. PXN, a cytoskeletal protein, when overexpressed, facilitated tumor progression and acted as an oncogene by regulating Bcl-2 [39]. In hepatocellular carcinoma, ITGB1 regulated the cell cycle process via the PXN/YWHAZ/AKT pathway, promoting hepatocellular carcinoma progression [40]. Overexpressing JUP, a homolog of β-catenin, reduced the expression of oncogenic STAB1 [41]. V-Crk avian sarcoma virus CT10 oncogene homolog-like (CRKL) acts as an adaptor protein of the Crk family and participates in cell proliferation, adhesion, and migration [42]. Moreover, CRKL regulates the activity of Ras, JNK, and Stat5 signaling pathways [43, 44]. CRK is overexpressed in various cancers, and its expression often correlates with tumor grade [45]. Arf6 belongs to the small GTPases ADP-ribosylation factor (Arf) family. Studies have established a significant correlation between the activation and high expression of Arf6 protein and the invasion and metastasis of several tumors. Inhibiting Arf6 activity could inhibit tumor invasion and metastasis [46, 47]. STAT3 serves as the convergence point of multiple cancer-related pathways and is frequently overactivated in cancer progression [48]. NF-κB1 is a major component of NF-κB. Many cancers are marked by the elevated activity of NF-κB, which serves as a survival factor for malignant cells due to its major anti-apoptotic function [49]. NF-κB and STAT3 cooperatively promote tumor development through functional interactions, inducing pro-tumor genes, including genes that generate anti-apoptotic chemokines and immunosuppressive cytokines [50–52]. IκB encodes IκB kinase β, which facilitates IκB degradation and mediates key steps in NF-κB activation [53, 54]. These hub proteins were intricately connected to tumor development and warrant further investigation in MD.

PRP^C^ (cellular prion protein) is encoded by PRNP. When it misfolds into PrP^Sc^ (scrapie isoform of prion protein) and accumulates in neuronal cells, it leads to prion disease [55]. Compared to adjacent non-tumor tissues, PrP^C^ expression was found to be upregulated in various cancer tissues, including colorectal cancer [56], gastric cancer [57], and pancreatic cancer [58]. Our laboratory confirmed the correlation between MDV infection and PRNP expression at the cellular level and in the tissues of naturally developed MD chickens [59, 60]. This result aligns with the present study. A transcriptomic study highlighted three differentially expressed genes, *TCRB*, *HSP70*, and *XCL1*, between MDV-infected resistant and susceptible chickens. Therefore, they represent key targets for future studies elucidating the mechanisms of MD resistance [61]. Our proteome results revealed that TCRB and HSP70 protein expression remained unchanged, while XCL1 expression was upregulated, consistent with a previous study [18]. Furthermore, several members of the HSP40 family (A3, A4, B6, C11, C13) also exhibited changes in this study.

Further analysis uncovered 40 unique DEPs at 45 dpi, mainly involved in cell adhesion (FN1), cell attachment (ITGA2, THBS1), signal transduction (LRP1, TGM2, RRAS2, GNAI2, RAB11B, RAB5B, CLTA), cytoskeleton (EZR), immunity (XCL1, CD79B), transcriptional regulation (TBL1X, PRKAA1, POLR2L, TCEB1), and apoptosis (DFFA, IL2RA). Among these proteins, THBS1 plays a vital role in tumorigenesis. KEGG analysis showed that THBS1 participates in PI3K-Akt, ECM-receptor interaction, microRNAs in cancer, and the Rap1 pathway. THBS1, a tumor-specific extracellular matrix protein and tumor suppressor, is known to inhibit angiogenesis, regulate anti-tumor immunity, and stimulate tumor cell migration in the tumor microenvironment [62]. In hepatocellular carcinoma cells, upregulation of THBS1 promoted cell proliferation and inhibited apoptosis [63]. In bladder urothelial carcinoma (BLCA), THBS1 counteracted the tumor-associated behavior of miR-19a-3p in BLCA cells [64]. This underscores THBS1 as an important target for tumor therapy, deserving further exploration. It is well-known that tumor cells primarily rely on glycolysis for their energy needs, whereas normal cells predominantly rely on aerobic oxidation. Consequently, glycolytic genes are often overexpressed in tumor cells. Additionally, the qPCR and proteomics results in this study demonstrated that the changes in FN1 and CTSD were consistent with previous transcriptome studies [17].

Many studies have found that PrP is overexpressed in various tumor tissues. This overexpression induces cell proliferation without limits, malignant differentiation, and variations. It affects tumor cell adhesion, invasion, diffusion, apoptosis, and other processes. Additionally, it influences multiple signaling pathways associated with tumor formation. Previous studies conducted in our laboratory revealed that the distribution of the *PRNP* gene in chicken embryos is identical to that in mammals. This suggests that PrP plays a crucial role in the development of chicken embryos and likely serves the same physiological functions in both chicken embryos and mammals [65]. Studies have indicated an upregulation of chicken PrP in the MD tumor cell line, MSB-1) [66]. In bovine mammary epithelial cells, the PrP protein expression increases within 12 hours of *Staphylococcus aureus* infection [67]. Earlier, the fluorescence-quantitative PCR method was used to prove the effect of PRNP expression on MDV infection at the cell level, indicating the relationship between the two. However, the specific mechanism remains unclear. In our study, we confirmed that the expression level of *PRNP* peaked at 30 and 45 dpi in the spleen, closely mirroring the expression trends of *meq* and *gB*.

## Conclusion

The study demonstrates that GX0101 causes significant damage to the spleen. Histopathological examination revealed severe damage to the spleen structure, marked reduction in white pulp lymphocytes, and necrosis of some lymphocytes in conjunction with tumor cell proliferation. The expression of MDV-related genes peaked at 45 dpi, along with the expression of PRNP. This positive correlation with MDV replication underscores the pivotal role of PRNP in MDV infection. However, the role of THBS1 in MD remains a subject for further investigation. Splenic proteome analysis identified DEPs associated with metabolic pathways related to inflammation, apoptosis, and tumors. This proteomics study lays the groundwork for subsequent research into the interaction between host proteins and MDV strain GX0101.

### Supplementary Information

**Additional file 1.**

## Data Availability

The datasets related to the current study are available at https://www.iprox.cn/page/home.html; iProX ID: IPX0006232002.
